# Mindfulness Meditation Can Benefit Glaucoma Patients

**DOI:** 10.5005/jp-journals-10078-1239

**Published:** 2019

**Authors:** Tanuj Dada, Meghal Gagrani

**Affiliations:** 1,2 Dr Rajendra Prasad Centre for Ophthalmic Sciences, All India Institute of Medical Sciences, New Delhi, India

## Abstract

**How to cite this article:** Dada T, Gagrani M. Mindfulness Meditation Can Benefit Glaucoma Patients J Curr Glaucoma Pract 2019; 13(1):1–2.

Glaucoma is a form of neurodegenerative disease characterized by loss of retinal ganglion cells. Glaucomatous damage involves trans-synaptic neurodegeneration affecting the visual pathway including the lateral geniculate nucleus and the occipital cortex.^1^

Various mechanisms^2–6^ have been implicated in neuronal apoptosis including barotrauma, hypoxia, glial cell activation, decrease in neurotrophins, central insulin resistance, mitochondrial dysfunction leading to oxidative damage,^2^ and glutamate excitotoxicity. In the light of these pathogenetic mechanisms leading to neurodegeneration, glaucoma has been recently labeled as type IV diabetes.”^3^ Raised intraocular pressure is the most frequently implicated risk factor in the progression of glaucoma and is currently the only target for therapeutic interventions.

Glaucoma is associated with a poor quality of life and patients have increased anxiety and depression due to the stress of this blinding disorder. Stress and glaucoma have a two-way relationship, each exacerbating the effect of the other. Stress leads to the release of endogenous cortisol which can lead to an increase in intraocular pressure (IOP) as glaucoma patients are high steroid responders. Like in any other chronic stressful conditions, serum cortisol levels are higher in patients with glaucoma and ocular hypertension. If we can reduce stress, we can reduce levels of endogenous cortisol and thereby potentially reduce IOP.

Various researchers have tried to decipher the role of the brain and use it as a potential therapeutic target in glaucoma.^7^ The key question is—Can meditation be used for this purpose?

**Flowchart 1:**
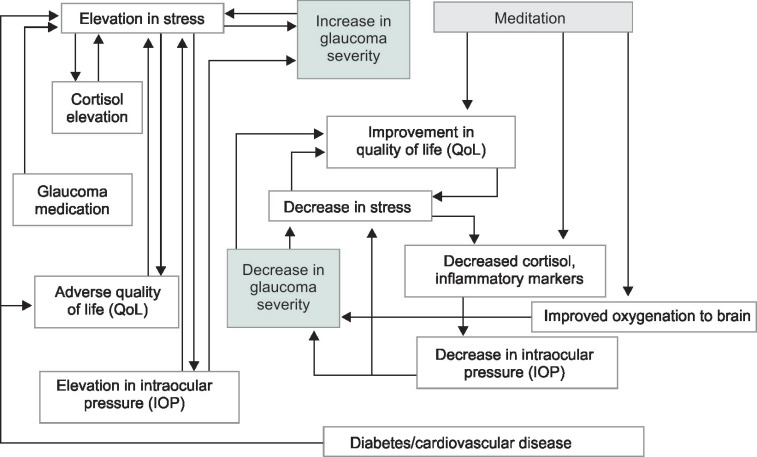
Impact of meditation in glaucoma patients

Meditation refers to a family of self-regulation practices that focus on training attention and awareness to bring mental processes under greater voluntary control and thereby foster general mental well-being and development and/or specific capacities such as calm, clarity, and concentration.^8^ During meditation, attention can be focused on a mantra, sound, or breath. Mindfulness meditation focused on the breath has an additional benefit as slow breathing activates the parasympathetic nervous system and counters the stress response.

The practice of meditation has not only been associated with electroencephalographic changes but also significant structural changes in the brain.^9^ Increased structural connectivity^10^ and white matter changes accounting for the neuroplasticity have been found in long-term meditators. Long-term meditators have been found to increase cortical thickness in the prefrontal cortex, anterior insula, thalamus, and hippocampus.^11,12^ Additionally, short-term meditation has also shown to induce white matter changes in the cingulate cortex.^13^ These changes provide evidence of cortical plasticity associated with meditation, occurring by changes in myelin density, axonal membrane integrity, axonal density, etc.^14^

Meditation has been found to improve the quality of life and decrease stress in various chronic diseases like diabetes, hypertension, major depression, and cancers. This stress reduction has also been associated with a reduction in the risk of mortality in cardiovascular disease.^15^ Meditation has been associated with a reduction in serum cortisol levels and plasma catecholamines.^16,17^ Additionally, it causes downregulation of proinflammatory gene expression and decreases oxidative stress with an improvement in mitochondrial function.^18,19^

How can meditation benefit patients with glaucoma? A short-term course of mindfulness meditation was found to reduce IOP, reduce stress biomarkers, and positively modulate gene expression with an improvement in the quality of life in patients with glaucoma.^20^ Additionally, a short-term course of meditation was found to improve the cerebral oxygenation of the prefrontal cortex in glaucoma patients using functional near-infrared spectroscopy,^21^ with an increase in brain-derived neurotrophic factor, suggesting a possible role in preventing retinal ganglion cell death.^21^

Another aspect of glaucoma which is often neglected is the caregiver burden. A significant burden of emotional and psychosocial stress is present in caregivers of glaucoma patients and caregivers also suffer from greater emotional and physical disorders as compared to noncaregivers. Meditation has been suggested as an effective strategy for stress reduction in caregivers also.^22–24^

In conclusion, meditation can positively modulate cellular pathways (Flowchart 1) involved in glaucoma pathogenesis leading to a significant reduction in IOP and improve the quality of life of glaucoma patients. It is a technique which involves a low risk and low cost and can be universally done by glaucoma patients, even if elderly or bedridden and can serve as a useful adjunct to standard ocular hypotensive therapy for glaucoma patients.
